# Regulated Cell Death in Lenvatinib Resistance of Hepatocellular Carcinoma: from Molecular Mechanisms to Therapeutic Strategies

**DOI:** 10.7150/ijbs.107195

**Published:** 2025-02-18

**Authors:** Ronggao Chen, Xin Hu, Yingchen Huang, Yao Jiang, Guanrong Chen, Qiaonan Shan, Xiao Xu, Shusen Zheng

**Affiliations:** 1 Division of Hepatobiliary and Pancreatic Surgery, Department of Surgery, First Affiliated Hospital, School of Medicine, Zhejiang University, Hangzhou 310003, China.; 2 NHC Key Laboratory of Combined Multi-organ Transplantation, Hangzhou 310003, China.; 3 Zhejiang University School of Medicine, Hangzhou 310058, China.; 4 The Fourth School of Clinical Medicine, Zhejiang Chinese Medical University, Hangzhou 310053, China.; 5 School of Clinical Medicine, Hangzhou Medical College, Hangzhou 310059, China.; 6 Institute of Translational Medicine, Zhejiang University, Hangzhou 310000, China.

**Keywords:** Lenvatinib, Hepatocellular carcinoma, Drug resistance, Combination treatments, Regulated cell death

## Abstract

Lenvatinib, a multi-target tyrosine kinase inhibitor (TKI), has been established as the first-line treatment for advanced hepatocellular carcinoma (HCC) because of its superior efficacy when in comparison with sorafenib. However, the inevitable development of drug resistance is a significant barrier to achieve a curative outcome and negatively impacts the prognosis. Therefore, it is imperative to delve into the mechanisms underlying lenvatinib resistance (LR) and to identify potential strategies for rational combination treatments. Regulated cell death (RCD) refers to the process by which cells undergo demise when the adaptive responses are insufficient to maintain homeostasis, and RCD takes a crucial part in the disease progression and response to therapeutic agents including TKI of cancer. Resisting cell death is one of the fundamental hallmarks and the major reasons contributing to drug resistance in cancer. Particularly, numerous studies have demonstrated that RCD (including apoptosis, autophagy, ferroptosis, cuproptosis and pyroptosis) plays a significant role in the emergence of LR in HCC. This article offers an in-depth review of recent discoveries concerning the mechanisms of LR in relation to RCD and proposes potential strategies to boost the effectiveness of lenvatinib by incorporating RCD modulators.

## Introduction

Hepatocellular carcinoma (HCC), the leading type of primary liver cancer, is the sixth most frequently diagnosed cancer and the third leading cause of cancer-related deaths worldwide 1. At present, the therapeutic options for HCC include liver transplantation (LT), surgical resection, radiofrequency ablation (RFA), transcatheter arterial chemoembolization (TACE), stereotactic body radiotherapy (SBRT), and systemic therapy. Up until now, LT, surgical resection and RFA are the only curative treatments available for HCC patients 2. Regrettably, the majority of HCC patients are usually diagnosed at advanced stages, making them ineligible for curative interventions 3, 4. Systemic therapy constitutes the mainstay of treatment for HCC patients who are in the advanced stages of the disease 5, 6. Systemic therapy for HCC encompasses conventional chemotherapy, targeted therapies specific to the disease, and immunotherapy 7. Kinase inhibitors are the preferred option for targeted therapy in HCC, and the first-line agents contain sorafenib and lenvatinib. In addition, new targets in HCC and their corresponding treatment approaches are continuously evolving, yet there remains significant progress needed before they can be effectively utilized in clinical settings 8, 9.

In 2007, sorafenib, a pioneering multi-target kinase inhibitor, received official approval for the treatment of advanced HCC. The approval was based on the SHARP trial, which showed a significant increase in progression-free survival (PFS) from 2.8 months to 5.5 months, as well as an improvement in overall survival (OS) from 7.9 months to 10.7 months 10. Despite the statistically significant results, the objective response rate (ORR) to sorafenib was a modest 2%, with the majority of patients experiencing only stable disease (SD) during the treatment period. In 2018, lenvatinib became the second first-line targeted therapy for advanced HCC to receive approval, based on the outcomes of the REFLECT trial 11. Lenvatinib demonstrated notable enhancements over sorafenib in secondary outcome measures, including a higher ORR, extended PFS, and a longer time to progression (TTP). Similar to sorafenib, lenvatinib is an oral small molecule tyrosine kinase inhibitor (TKI) that exhibits its anti-cancer effects by inhibiting a variety of targets, such as vascular endothelial growth factor receptor (VEGFR) 1-3, fibroblast growth factor receptor (FGFR) 1-4, platelet-derived growth factor receptor α, KIT, and RET 12. While the introduction of lenvatinib has led to promising progress in the treatment of advanced HCC, it has only resulted in an average survival extension of 2 to 3 months 13. Drug resistance significantly diminishes the clinical effectiveness of lenvatinib. Therefore, it is imperative to investigate the mechanisms underlying lenvatinib resistance (LR) and to identify potential targets for rational combination therapies. Studies on LR in HCC have identified potential mechanisms such as dysfunctional pathway activation, drug transport variations, regulated cell death (RCD), epigenetic alterations, influences from the tumor microenvironment (TME), the role of cancer stem cells (CSC), epithelial-mesenchymal transition (EMT), and others 14. Our previous study revealed that phosphorylated non-muscle myosin heavy chain 9 (p-MYH9) at Ser1943, could deubiquitinate and stabilize HIF-1α by recruiting (ubiquitin-specific protease 22) USP22 in HCC, which led to the development of LR 15. The employment of a casein kinase-2 (CK2) inhibitor alongside a USP22 inhibitor successfully overcame LR, suggesting a promising treatment strategy for HCC patients who experienced LR.

**Figure 1:**
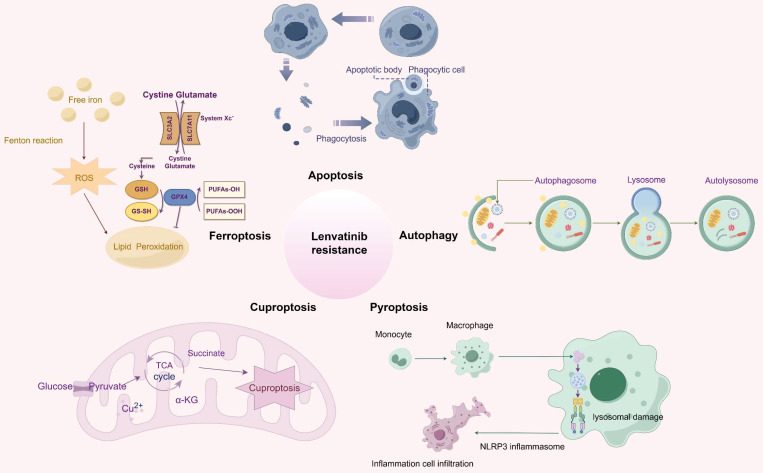
An overview of the molecular mechanisms of RCD in relation to LR. Apoptosis is the most renowned form of RCD characterized by the formation of apoptotic bodies; Autophagy refers to the process in which intracellular components including proteins and organelles are conveyed to lysosomes, degraded and recycled; Ferroptosis, driven by iron-dependent phospholipid peroxidation, leads to inactivation of GPX4 or system xc-cystine/glutamate antiporter, and producing excessive ROS; Cuproptosis is triggered by excessive Cu2+ ions, which bind to TCA cycle proteins, leading to mitochondrial aggregation; Pyroptosis is characterized by its lytic and inflammatory nature, typically initiated by inflammasomes, marked by cellular swelling, membrane rupture, and the release of intracellular contents. Abbreviations: LR: lenvatinib resistance; RCD: regulated cell death; ROS: reactive oxygen species; TCA: tricarboxylic acid.

RCD refers to the process by which cells undergo demise as a result of molecular mechanisms triggered by either intracellular or extracellular factors, and this occurs when the cell's adaptive responses are insufficient to maintain homeostasis 16. RCD encompasses a spectrum of distinct forms of cell demise, each characterized by unique mechanisms, including apoptosis, autophagy, ferroptosis, cuproptosis, pyroptosis, necroptosis, parthanatos, lysozincrosis and disulfidptosis 17. RCD plays a crucial role in the development of organisms and the maintenance of homeostasis, and various lethal subroutines in the process of RCD affect disease progression and response to therapeutic agents in cancer 18, 19. Resisting cell death is one of the several fundamental hallmarks and the major reasons contributing to drug resistance in cancer 20, 21. In particular, extensive studies have shown that RCD is involved in the development of resistance to lenvatinib in HCC (Figure 1). This research provides a comprehensive overview of the latest findings regarding the mechanisms underlying LR in the context of RCD, and proposes potential strategies aimed at enhancing the efficacy of lenvatinib with RCD modulators.

## Apoptosis

Apoptosis, widely recognized as the most renowned form of regulated cell death, functions as a critical physiological process that curbs the cell proliferation 22. It plays a dual role in either preserving tissue homeostasis or eliminating cells that could pose a threat to the organism 23. Efficient cancer therapies frequently target the regulation of apoptotic signaling pathways, and blocking these pathways may result in resistance to anti-cancer treatments 24, 25. Up until now, extensive researches have indicated that apoptosis signaling is crucial for triggering LR in HCC, and therefore targeting the pathways involved in apoptosis might serve as a potential approach to reverse LR.

Zhao *et al.* reported that overexpressed fibroblast FGFR1, along with the activation of its downstream pathways AKT/mTOR and ERK, could cause LR by inhibiting apoptosis in HCC 26. Tan's study revealed that glutathione peroxidase (GPX) 2 exerted a significant role in reactive oxygen species (ROS) related apoptosis induced by lenvatinib in HCC cells, and over-expression of GPX2 could induce LR through impairing apoptosis by acting as a downstream gene of β-catenin 27. NAD(P)H:quinone oxidoreductase 1 (NQO1) was up-regulated in LR HCC cells, and high levels of NQO1 suppressed the ROS-associated apoptosis induced by lenvatinib, which led to LR 28.

Previous researches have pinpointed certain compounds that possess the ability to modulate pathways associated with apoptosis. Among these compounds are members of the Bcl-2 protein family, caspase family, c-Myc, tumor necrosis factor-related ligands and their receptors, Apaf-1, Cytochrome C, the NF-κB signaling, and the p53 protein. Genetic alterations or the activation of specific pathways can suppress the process of apoptosis and foster resistance to lenvatinib by modifying the behavior of these molecular components. Yu *et al.* demonstrated that HCC cells with high levels of lncMT1JP expression could acquire resistance to lenvatinib via triggering the inhibition of apoptosis 29. The underlying mechanism involved lncMT1JP modulating the miR-24-3p/BCL2L2 pathway, which resulted in the suppression of apoptosis. Xu's study showed that down-regulation of miR-128-3p contributed to LR through inhibiting apoptosis by attenuating the cleavage of caspase-9 and caspase-3 30. Mechanistically, miR-128-3p negatively regulated the expression of c-Met by binding to the 3'-UTR of c-Met mRNA, and resulted in activation of AKT pathway. Guo *et al.* reported that elevated levels of interferon regulatory factor 2 (IRF2) were linked to LR in HCC through the modulation of the Wnt/β-catenin signaling, which in turn affected the regulation of proteins involved in apoptosis (e.g., Bcl-2 and caspase-3) 31. Another study proved that the expressions of USP22 and Jumonji domain-containing protein 8 (JMJD8) were elevated in HCC cells resistant to lenvatinib, and USP22 and JMJD8 formed a regulatory axis that regulated LR by altering apoptosis-related protein c-Myc 32.

Methylation, a significant epigenetic mechanism, encompasses the enzymatic process of transferring methyl group from active methyl donors to various other molecules, including DNA, RNA and histones 33-35. Abnormal methylation could result in progression and drug resistance in HCC 36. Methylation modifications of RNA mainly include m6A, m7G, m5C, m1A 37. m7G modification, prevalent in the 5' caps of eukaryotic mRNA or within rRNA and tRNA across all species, is linked to tumorigenesis and multiple cancer-related biological behaviors 38. Recently, Huang *et al.* reported that the m7G tRNA modification, catalyzed by the methyltransferase-like protein (METTL) 1, specifically modulated the EGFR signaling pathway, enhancing LR via impairing the apoptosis capacity of HCC cells 39. Given that EGFR is a downstream target of METTL3, Wang *et al.* discovered that METTL3 was identified as the most notably increased protein among these m6A regulators in HCC cells that are resistant to lenvatinib, and inhibition of METTL3 increased cell apoptosis upon lenvatinib treatment, which overrode LR 40. In addition, they found that LR HCC patients exhibit elevated levels of METTL3, and patients with reduced METTL3 levels had better survival outcomes. N4-acetocytidine (ac4C), an mRNA acetylation modification, is implicated in the pathogenesis of various human diseases, including cancer 41. Pan's research demonstrated that NAT10-mediated mRNA ac4C modification of HSP90AA1 regulated tumor metastasis and LR in HCC 42. Thus, identifying new therapeutic targets in the realm of translational regulation holds great promise for improving the prognosis of individuals with HCC.

Accordingly, deepening our comprehension of the link between apoptosis and LR is of significant clinical relevance, particularly for strategies aimed at reducing drug resistance.

## Autophagy

Autophagy refers to the process in which intracellular components including proteins and organelles are conveyed to lysosomes, degraded and recycled, thereby meeting the metabolic demands and supporting the self-renewal of cells 43. Various cancer treatments can trigger autophagy, and enhanced autophagy acts as an acquired resistance mechanism during exposition to stresses encountered from metabolic demands and therapeutic interventions 44. For instance, in prostate cancer, breast cancer and gastrointestinal stromal tumors, inhibiting autophagy has been shown to overcome resistance to chemotherapy drugs 45-47. To date, research indicates that autophagy is crucially involved in the development of LR in HCC.

**Figure 2:**
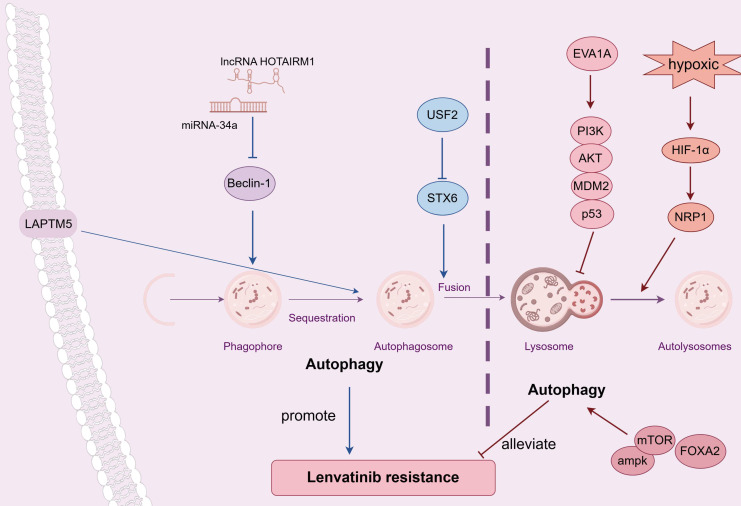
Dual effect of autophagy on LR. LAPTM5 could enhance the intrinsic autophagic flux, which contributed to LR; Silencing TMX2 relieved LR by suppressing autophagy through KPNB1/TFEB pathway; STX6, negatively regulated by USF2, accelerated autophagic flux, which led to LR; The suppression of lncRNA HOTAIRM1 led to a reduction in autophagy levels via miR-34a/Beclin-1 axis, which counteracted LR. FOXA2 overexpression impared LR by upregulating the AMPK-mTOR-autophagy pathway; NRP1, upregulated by HIF-1α in hypoxic conditions, could amplify LR by diminishing autophagy; EVA1A loss drove LR via inhibiting autophagy through suppressing PI3K/AKT/MDM2/p53 signaling. Abbreviations: LR: lenvatinib resistance; LAPTM5: lysosomal protein transmembrane 5; TMX2: thioredoxin related transmembrane protein 2; STX6: Syntaxin-6; USF2: upstream stimulatory factor 2; FOXA2: forkhead box protein A2; NRP1: Neuropilin-1; EVA1A: Eva-1 homolog A.

Autophagy exhibits a dual-function in modulating the drug resistance of lenvatinib in HCC (Figure 2). Using CRISPR-Cas9 screen along with database analysis, Pan *et al.* pinpointed lysosomal protein transmembrane 5 (LAPTM5) as a crucial contributor to LR in HCC, and discovered that LAPTM5 could enhance the intrinsic autophagic flux by promoting the formation of autolysosomes 48. Importantly, in clinical HCC samples, lenvatinib-sensitive patients showed lower LAPTM5 levels than LR patients, and recurrent patients after surgery had higher LAPTM5 expressions than non-recurrent ones. Similar results were obtained in various studies which demonstrated up-regulated autophagy may promote LR in HCC. Syntaxin-6 (STX6), negatively regulated by the upstream stimulatory factor 2 (USF2), facilitated the fusion of autophagosomes with lysosomes and subsequently accelerated autophagic flux, which led to LR 49. Gu's study indicated that the suppression of the long non-coding RNA HOTAIRM1 led to a reduction in autophagy levels within lenvatinib-resistant HCC cells via miR-34a-Beclin-1 regulatory axis, and resulted in an enhanced responsiveness to lenvatinib treatment 50. Moreover, the expression of HOTAIRM1 was considerably elevated in HCC patients resistant to lenvatinib compared to those who were sensitive to the agent. However, contradictory findings were obtained by other researches, showing through experiments that increasing autophagy could potentially boost the effectiveness of lenvatinib and reduce LR in HCC. Eva-1 homolog A (EVA1A) loss, which prevented the autophagosome formation, was proved to drive LR via inhibiting mutant p53 degradation through suppressing the PI3K/AKT signaling pathway 51. Palanca's study indicated that Neuropilin-1 (NRP1), upregulated by HIF-1α in hypoxic environments, could negate the therapeutic impact of lenvatinib, resulting in LR. This occured because lenvatinib's antitumor efficacy was mediated by the autophagy-dependent degradation of NRP1, which involved the assembling of autolysosome 52. Wang *et al.* reported that forkhead box protein A2 (FOXA2) overexpression was found to amplify the effectiveness of lenvatinib on HCC cells by upregulating the AMPK-mTOR-autophagy signaling pathway 53. Further investigation is warranted to explore the potential of modulating autophagy, either through inhibition or enhancement, as a strategy to potentiate the antitumor effects of lenvatinib in HCC treatment.

Autophagy is categorized into selective and non-selective forms based on its substrate degradation mechanism 54. In selective autophagy, specific autophagic pathways are employed to target and recycle unwanted or damaged cellular components such as mitochondria, ribosomes, peroxisomes, lysosomes, endoplasmic reticulum (ER), nuclei, lipid droplets and proteasomes. Impairments in selective autophagy are intimately associated with a range of pathologies, including cancer, metabolic disorders, neurodegenerative diseases and heart failure 55-58. Mitochondrial autophagy (i.e., mitophagy), a specialized type of selective autophagy, targets and removes impaired mitochondria to maintain the integrity of the mitochondrial network 59. Studies have shown that mitophagy takes a vital part in regulating LR in HCC. CRISPR activation screen was applied in Zhang's study, and they identified TMX2 (thioredoxin related transmembrane protein 2) as an essential gene for HCC cell survival 60. Further experiments found that TMX2 relieved LR and amplified the anti-tumor impact of lenvatinib in HCC by suppressing autophagy and mitophagy, which was achieved through the inhibition of karyopherin subunit beta (KPNB) 1's nuclear export and transcription factor EB (TFEB) 's nuclear import. LINC01607, was proved to act as a competitive endogenous RNA (ceRNA), antagonize miR-892b and thereby stimulate the upregulation of P62. The increase in P62 activity induced protective mitophagy, contributing to the development of LR in HCC 61. Zheng's research revealed that under cellular stress, Stomatin-like protein 2 (STOML2) was capable of interacting with and stabilizing PTEN-induced putative kinase 1 (PINK1), which significantly amplified the PINK1-Parkin-mediated mitophagy process in HCC. This enhancement of mitophagy was identified as a key factor contributing to the development of LR 62. Wang *et al.* reported that in lenvatinib-resistant HCC cells, the excessive activation of BCL2 interacting protein 3 (BNIP3)-driven mitophagy enhanced glycolytic flux, resulting in constantly sustained competitive edge of lenvatinib-resistant cells over their sensitive counterparts, which maintained LR 63. This was achieved by shifting energy metabolism from mitochondrial oxidative phosphorylation to glycolysis, which was regulated through the AMPK-enolase 2 (ENO2) signaling pathway. In forthcoming studies, it will be crucial to investigate the strategies targeting selective autophagy with the aim of amplifying the therapeutic effects of lenvatinib. Simultaneously, ensuring that the essential functions of autophagy within the cellular context are preserved will be of paramount importance to avoid unintended side effects.

## Ferroptosis

The study of ferroptosis has experienced a dramatic surge in interest and development since the concept was first introduced in 2012. This distinctive form of cell death, propelled by the peroxidation of phospholipids that is reliant on iron, is governed by a variety of cellular metabolic processes, which include the maintenance of redox balance, the management of iron levels, the functioning of mitochondria, and the metabolism of amino acids, sugars and lipids 64, 65. Furthermore, it is influenced by numerous signaling pathways that are pertinent to various diseases, such as cancers, autoimmune diseases, cardiovascular diseases, chronic liver diseases and metabolic diseases 66-70. Intriguingly, cancer cells that are resistant to therapy, especially those in a mesenchymal state and inclined to spread, exhibit a remarkable susceptibility to ferroptosis 71-73. So far, studies have shown that ferroptosis plays a significant role in the induction of LR in HCC.

It was reported that lenvatinib might trigger ferroptosis by suppressing the FGFR/system xc-/GPX4 pathway 74. An increased expression of the nuclear factor erythrocyte 2-related factor 2 (Nrf2), a crucial transcription factor for regulating intracellular ROS, could suppress lenvatinib-induced ferroptosis and cause LR in HCC. Additionally, low FGFR4 expression and high P-Nrf2 expression in HCC tissues are associated with LR and shorter PFS. Zeng's study showed that serine beta-lactamase-like protein (LACTB) was identified as a downstream target of lenvatinib, and overexpression of LACTB triggered ferroptosis through (solute carrier family 7 member 11) SLC7A11/GSH/GPX4 signalling pathway, resulting in the enhancement of lenvatinib's anti-tumor efficacy and attenuation of LR 75. Lai *et al.* reported that heme oxygenase-1 (HO-1) knockdown up-regulated GPX4 in HCC, leading to reduction of ferroptosis and LR 76. Another study revealed that GPX4 transcriptionally inhibited ferroptosis via grainyhead-like transcription factor 3 (GRHL3)/PTEN/PI3K/AKT axis, which accentuated LR 77. Hiromatsu *et al.* reported that transferrin receptor (TR) knockdown abolished lenvatinib-induced ferroptosis, which contributed to the development of LR in HCC cell lines 78. Hypoxia is a common characteristic of the TME, and it is linked to the pathological features, prognosis, and therapeutic outcomes in HCC patients 79. Peroxisome proliferator-activated receptor-gamma coactivator-1α (PPARGC1A) was down-regulated in lenvatinib-resistant HCC cells, and under hypoxia, overexpression of PPARGC1A improved sensitivity to ferroptosis and counteracted LR by controlling bone morphogenetic protein and activin membrane-bound inhibitor (BAMBI)/ACSL5 signaling 80. PPARGC1A/BAMBI/ACLS5 pathway was hypoxia-responsive and WTAP-mediated m6A modification modulated PPARGC1A mRNA under hypoxic conditions.

Noncoding RNAs, encompassing microRNAs (miRNAs), long noncoding RNAs (lncRNAs), and circular RNAs (circRNAs), have been implicated in the biological processes associated with ferroptosis and the development of drug resistance in a range of cancers 81, 82. lncRNA HAND2-AS1 was down-regulated in lenvatinib-resistant HCC cell lines, and overexpressed HAND2-AS1 promoted ferroptosis to reverse LR by competing endogenous miR-219a-1-3p in HCC cells 83. In recent developments, it has been found that there is cross-signaling between ferroptosis and selective autophagy, with ferroptosis being shown to be heavily reliant on selective autophagy, such as ER-phagy, lipophagy and ferritinophagy 84. Bi's research uncovered that ER-phagy-driven ferroptosis was involved in HCC cells treated with lenvatinib, and they discovered that the reduction of circFAM134B specifically directed ER-phagy to enhance ferroptosis induced by lenvatinib 85. Mechanically, circFAM134B competitively interacted with poly (A) binding protein cytoplasmic 4 (PABPC4), consequently affecting the nonsense decay of family with sequence similarity 134, member B (FAM134B) mRNA. Zhang's study demonstrated that the overexpression of circPIAS1 suppressed ferroptosis by sponging mir455-3p, which in turn led to the upregulation of Nuclear Protein 1 (NUPR1). Additionally, NUPR1 facilitated the transcription of ferritin heavy chain 1 (FTH1), increasing iron storage within HCC cells and thus providing resistance to ferroptosis and lenvatinib treatment 86.

In conclusion, there is a strong association between ferroptosis and the development of resistance to lenvatinib. Further investigation into new mechanisms and potential countermeasures is warranted.

## Other forms of RCD

### Cuproptosis

Cuproptosis represents a novel type of regulated cell death that is activated by an overabundance of Cu^2+^ ions 87. Intracellular copper targets and attaches to lipoylated elements in the tricarboxylic acid (TCA) cycle. The clumping of copper-bound lipoylated proteins in the mitochondria, along with the subsequent decrease in iron-sulfur cluster, induce protein misfolding stress and eventual cell death 88. Cuproptosis has been garnering increasing attention, particularly after its link to cancer was established, prompting many researchers to delve into the connection between cuproptosis and various types of cancer 89, 90. Yang *et al.* reported that cuproptosis-related gene Dihydrolipoamide S-acetyltransferase (DLAT) was up-regulated in the drug-resistant group treated with lenvatinib, and the mechanism study showed that DLAT might trigger LR by inhibiting cuproptosis 91. Li's study showed that a decrease in the expression of anti-cuproptotic proteins (DLAT, LIAS, and FDX1) and an increase in the expression of the pro-cuproptotic protein CDKN2A were observed in LR HCC cells, indicating cuproptosis took a vital part in drug resistance of lenvatinib 92.

### Pyroptosis

Pyroptosis is a form of regulated cell death characterized by its lytic and inflammatory nature, typically initiated by inflammasomes and carried out by gasdermin proteins 93. Key features of pyroptosis include cellular swelling, the breaching of the cell membrane, and the subsequent release of intracellular contents 94. Appealingly, pyroptosis, being a form of immunogenic cell death, offers a novel approach to cancer elimination by triggering pyroptotic cell demise and stimulating a robust antitumor immune response 95, 96. Fu's research reported that treatment with lenvatinib increased the mRNA and protein levels of pyroptosis-related gene GSDME, and up-regulated active GSDME N-terminal in HCC cell lines, implying that down-regulation of GSDME might induce LR by inhibiting pyroptosis 97.

## Strategies to overcome LR with RCD modulators

The overall response rate of lenvatinib in HCC patients is approximately 40%, yet its clinical utility is frequently constrained by the development of drug resistance. Therefore, there is an imperative to enhance the potency of lenvatinib in clinical practice. Up to this point, we have explored various pathways and cellular mechanisms that contribute to the development of drug resistance to lenvatinib within the realm of RCD. We further provide a summary of the potential strategies to overcome LR with RCD modulators (Table 1).

### Targeting apoptosis

**Figure 3:**
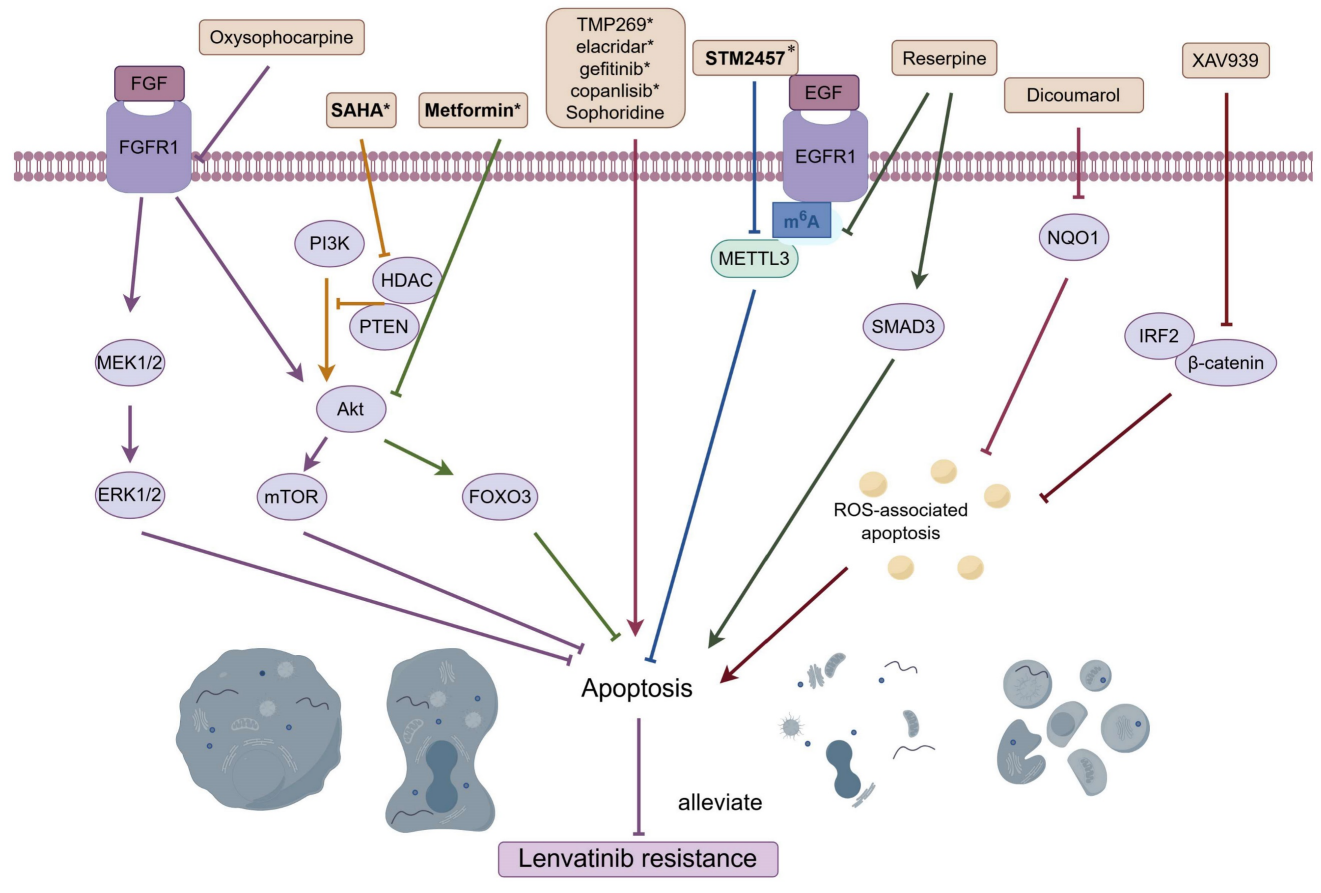
Combined treatments that reverse LR by targeting apoptosis. Oxysophocarpine vanquished LR by promoting apoptosis via FGFR1 and its downstream signaling; Dicoumarol, the NQO1 inhibitor, counteracted LR by enhancing ROS-associated apoptosis; Inhibiting β-catenin with the use of XAV-939 overpowered LR via fostering apoptosis; Metformin weakened LR by the promotion of apoptosis through the modulation of the AKT-FOXO3 pathway; SAHA, an HDAC inhibitor, mitigated LR by triggering cell apoptosis via modulating the PTEN/AKT pathway; METTL3 inhibitor STM2457 surmounted LR via increasing cell apoptosis through METTL3-m6A-EGFR-axis; Reserpine reversed LR by inducing apoptosis through the suppression of m6A and the activation of SMAD3; TMP269, Sophoridine, elacridar, gefitinib and copanlisib, were all proven to ameliorate LR via regulating apoptosis-related pathway. Abbreviations: LR: lenvatinib resistance; FGFR1: fibroblast growth factor receptor; NQO1: NAD(P)H:quinone oxidoreductase 1; ROS: reactive oxygen species; METTL3: methyltransferase-like protein 3. Medications that exhibit a synergistic effect with lenvatinib are indicated by an asterisk (*).

Targeting apoptosis is a promising way to overcome LR (Figure 3). Oxysophocarpine, an alkaloid derived from the plant *Siphocampylus verticillatus*, possesses a range of therapeutic properties, including anti-tumor effects, anti-inflammatory capabilities, and antiviral activities 98-100. As mentioned earlier, overexpressed FGFR1 together with activated AKT/mTOR and ERK pathways could induce LR in HCC, and oxysophocarpine could reduce the expression of FGFR1 and concurrently suppress the downstream signaling, thereby enhancing the sensitivity of lenvatinib 26. High levels of NQO1 contributed to LR by suppressing ROS-associated apoptosis, and the concurrent administration of lenvatinib with the NQO1 inhibitor, dicoumarol, counteracted drug resistance 28. IRF2/β-catenin pathway was stimulated in HCC LR cells, and inhibiting β-catenin with the use of XAV-939 effectively significantly enhanced the efficacy of lenvatinib by promoting cell apoptosis 31. Cheng *et al.* reported that metformin, a commonly utilized medication for diabetes, worked synergistically to boost the antitumor effects of lenvatinib in HCC by promoting apoptosis and triggering cell cycle arrest through the modulation of the AKT-Forkhead box protein O3 (FOXO3) pathway 101. Yan's research demonstrated the combined use of lenvatinib and SAHA, an HDAC inhibitor, works synergistically to suppress HCC cell proliferation and to trigger cell apoptosis by modulating the PTEN/AKT pathway 102. Moreover, Class IIa HDACI 103, Sophoridine 104, elacridar, gefitinib and copanlisib 105, were proven to ameliorate LR via regulating apoptosis-related pathway in HCC.

Increasing evidence has suggested that m6A methylation influences the stem-like properties of cancer cells and their resistance to a range of treatments, encompassing chemotherapeutic agents and targeted drugs 106. As noted before, METTL3-m6A-EGFR-axis was responsible for acquired resistant to lenvatinib, and the particular METTL3 inhibitor STM2457 amplified tumor response to lenvatinib in HCC animal models via increasing cell apoptosis 40. Another study reported that LR in HCC was associated with elevated levels of m6A and increased METTL3 expression, and the plant-derived hypertension drug Reserpine could potentially revert a resistant phenotype to a sensitive one by inducing apoptosis through the suppression of m6A and the activation of SMAD3 107.

### Targeting autophagy

**Figure 4:**
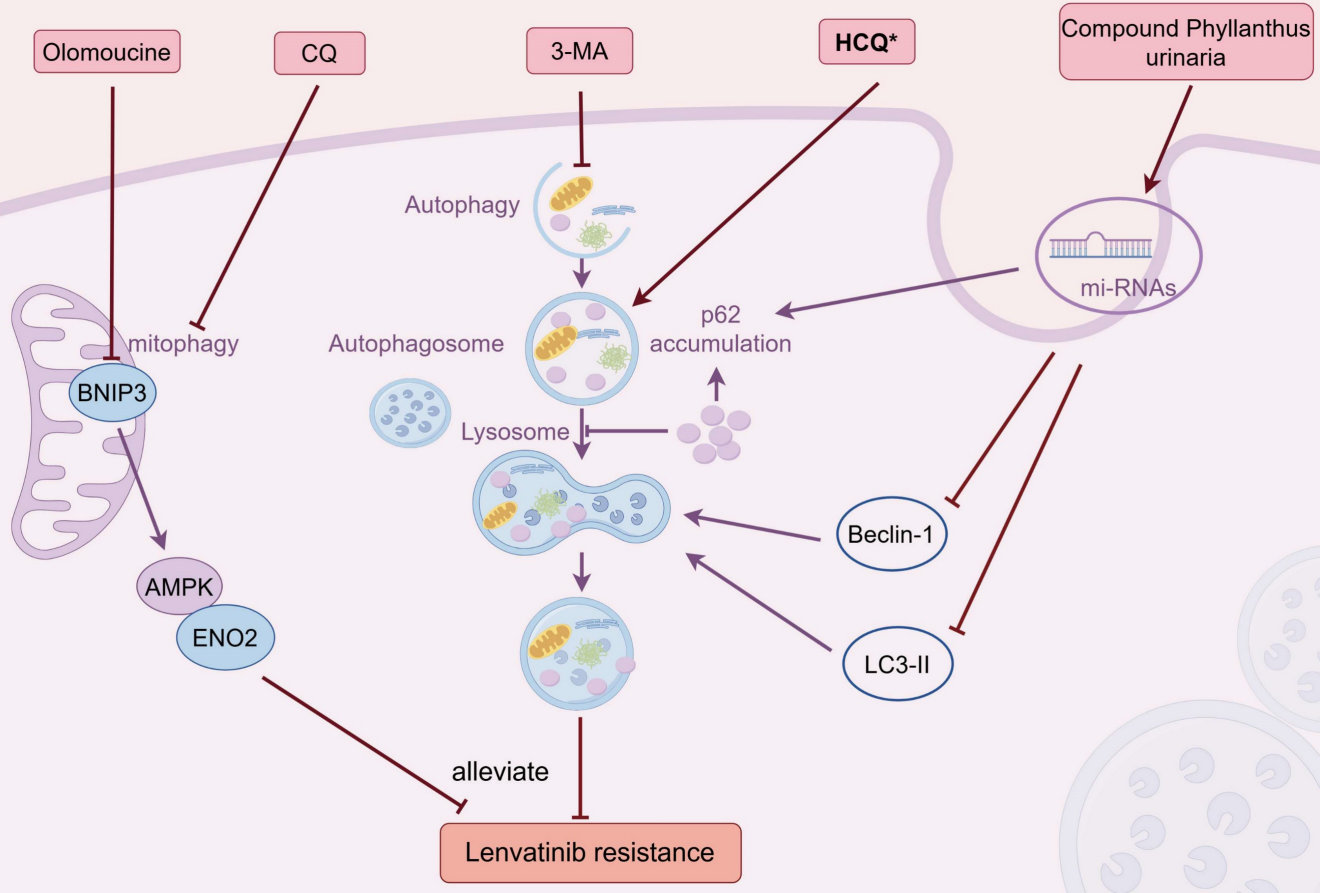
Combination therapies that overcome LR by targeting autophagy. Combined with HCQ or CQ counteracted LR by inhibiting autophagy or mitophagy, respectively; Concurrent administration of 3-MA surmounted LR through suppressing autophagy; Olomoucine reversed LR by inhibiting mitophagy via BNIP3-AMPK-ENO2 pathway; CP mitigated LR by modulating exosomal autophagy microRNAs and altering Beclin-1, LC3-II, and P62 levels. Abbreviations: LR: lenvatinib resistance; HCQ: hydroxychloroquine; CQ: chloroquine; 3-MA: 3-Methyladenine; CP: Compound Phyllanthus urinaria. Medications that exhibit a synergistic effect with lenvatinib are indicated by an asterisk (*).

Targeting autophagy represents a potential strategy to conquer LR (Figure 4). Hydroxychloroquine (HCQ) and chloroquine (CQ) are the sole pharmaceuticals currently accessible for clinical use that specifically aim to modulate autophagy 108. In LR HCC cells, HCQ combined with lenvatinib could work synergistically to suppress tumor growth and restore the sensitivity of lenvatinib by inhibiting the process of autophagy 48. Another study showed that blocking mitophagy with CQ sensitized HCC cells to lenvatinib treatment, which reversed LR 62.

Currently, an array of small molecule autophagy inhibitors has been crafted specifically for the purposes of scientific investigation, including 3-Methyladenine (3-MA). The concurrent administration of 3-MA heightened the effectiveness of lenvatinib and surmounted LR in HCC cells through autophagy modulation 50. Furthermore, several other agents were reported to conquer the drug resistance of lenvatinib via altering autophagy pathway. As mentioned before, the BNIP3-AMPK-ENO2 signaling pathway played a crucial role in sustaining the competitive advantage of LR HCC cells via regulating mitophagy-driven energy metabolism reprogramming, and co-treatment of BNIP3 inhibiter olomoucine substantially enhanced the antitumor effectiveness of lenvatinib 63. Compound *Phyllanthus urinaria* (CP), a traditional Chinese medicine (TCM), is effective across the spectrum of liver disease treatment, from viral hepatitis to liver fibrosis/cirrhosis and HCC, showcasing significant therapeutic potential 109, 110. Liao *et al.* reported that the synergistic administration of CP and lenvatinib potently suppressed HCC and counteracted LR by regulating autophagy-associated microRNAs in the exosomes, which is achieved by modulating the expression levels of Beclin-1, LC3-II, and P62 111. Icaritin, another TCM and derived from *epimedium* genus, has been documented as a potent anticancer substance against a spectrum of cancers, including HCC 112, 113. In a Phase I clinical trial, the safety profiles and initial evidence of sustained survival benefits of icaritin were observed in patients with advanced HCC, potentially attributed to its immunomodulatory effects 114. Of the 15 patients who were assessed, 7 (which is 46.7%) showed a positive outcome, including 1 showing partial response (PR, 6.7%) and 6 cases of SD (40%). The median TTP was calculated as 141 days (range: 20-343 days), and the median OS was estimated to be 192 days. Yu's study demonstrated that the targeted codelivery of icaritin and doxorubicin via PLGA-PEG-AEAA nanoparticles, in conjunction with lenvatinib, has been found to exert a synergistic effect by enhancing both mitophagy and apoptosis to provoke immunogenic cell death (ICD) in advanced HCC 115.

### Targeting ferroptosis

**Figure 5:**
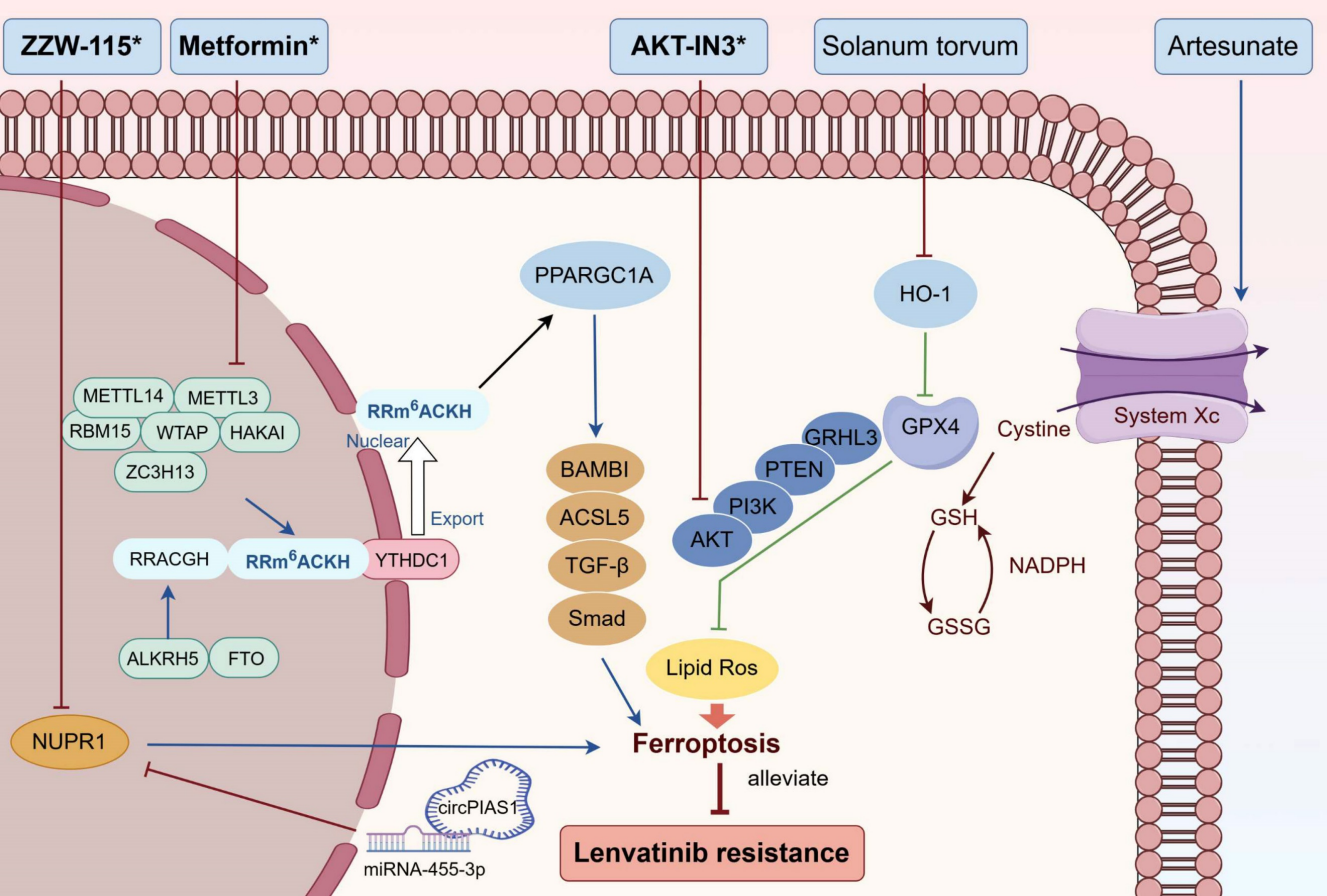
Combined treatments that combate LR by targeting ferroptosis. Metformin diminished LR by promoting ferroptosis via METTL3/ PPARGC1A/BAMBI/ACSL5 axis; ZZW-115 reversed LR by enhancing ferroptosis through circPIAS1/miR-4553p/NUPR1 pathway; AKT-IN3 conquered LR by improving ferroptosis via GPX4/GRHL3/PTEN/PI3K/AKT signalling; Solanum torvum weakened LR by boosting ferroptosis through the HO-1/GPX4 pathway; Artesunate suppressed LR through the promotion of ferroptosis. Abbreviations: LR: lenvatinib resistance; PPARGC1A: Peroxisome proliferator-activated receptor-gamma coactivator-1α; HO-1: heme oxygenase-1. Medications that exhibit a synergistic effect with lenvatinib are indicated by an asterisk (*).

Targeting ferroptosis emerges as a feasible approach to triumph over LR (Figure 5). As previously stated, overexpression of PPARGC1A counteracted LR by promoting ferroptosis, and metformin combated LR by restoring the expression of PPARGC1A and diminishing its m6A methylation through the suppression of METTL3 80. The circPIAS1/miR-455-3p/NUPR1 pathway has been documented to regulate LR by influencing ferroptosis. The administration of ZZW-115, an inhibitor of NUPR1, reversed the proliferative impact of circPIAS1 and enhanced the sensitivity of HCC cells to lenvatinib 86. GPX4/GRHL3/PTEN/PI3K/AKT axis was crucial in lenvatinib induced ferroptosis, and AKT-IN3, the AKT inhibitor, synergized with lenvatinib to reduce HCC metastasis 77. Another study showed that HO-1 knockdown up-regulated GPX4 in HCC, leading to reduction of ferroptosis and LR. The combination of *Solanum torvum* and lenvatinib demonstrated an additive effect by advancing ferroptosis through the HO-1/GPX4 pathway 76. Artesunate, a drug used to treat malaria, was found to enhance the ferroptosis-triggering effects of lenvatinib in HCC cell lines 78.

### Targeting cuproptosis

Disulfiram (DSF), a medication used for over sixty years to treat alcohol dependence, has demonstrated established pharmacokinetic properties, safety, and tolerability at dosages recommended by the US Food and Drug Administration (FDA) 116. Currently, DSF has attracted attention due to its potential to counteract drug resistance in cancer therapy 117. Li *et al.* reported that DSF, acting as a copper ionophore, was capable of coordinating with Cu^2+^ to combat LR in HCC by suppressing cuproptosis and cancer cell stemness 92. To mitigate systemic toxicity, DSF and CuO nanoparticles were encapsulated together to create an oil-in-water Pickering emulsion, which then merged with sodium alginate, leading to the formation of a DSF@CuO Gel through in situ gelation with Ca^2+^.

## Summary and Future Perspectives

Although lenvatinib demonstrates considerable therapeutic benefits, many patients eventually develop resistance to the medication, resulting in cancer progression and poor prognosis. In this review, we have conducted a thorough analysis of the pathways and cellular mechanisms that contribute to the onset of LR within the framework of RCD in HCC. Moreover, we additionally offer an overview of potential approaches to counteract LR by targeting RCD-associated pathways, utilizing either combined therapeutic strategies or nanotechnology-aided drug delivery systems.

In our review, it was demonstrated that while autophagy exhibited a dual-function in modulating the drug resistance of lenvatinib, the disruption of apoptosis, ferroptosis, cuproptosis and pyroptosis all contributed to the development of LR. Generally, up-regulation of autophagy, an adaption mechanism for tumor cells to survival under stresses encountered from metabolic demands and therapeutic interventions, promotes LR in HCC 48-50. However, modulating the autophagy of particular molecules or pathways might augment lenvatinib sensitivity. For instance, FOXA2 overexpression triggered the AMPK-mTOR axis, which resulted in activation of autophagy and attenuation of LR 53. Additionally, promoting the autophagy-dependent degradation of NRP1 52 or mutant p53 51 could potentially counteract LR. Given the autophagy's dualistic influence on LR, forthcoming studies must comprehensively consider the choice of experimental methodologies and animal models to ensure the acquisition of precise and dependable outcomes. According to the existing literature, inhibition of mitophagy sensitized HCC cells to lenvatinib treatment and overcomed LR 60-63.

Over the past decade, cutting-edge scientific methods, such as single-cell RNA sequencing (scRNA-seq), artificial intelligence (AI) models, and gene editing technologies like CRISPR have revolutionized biomedical research, offering novel approaches to dissect various aspects of tumor biology. Utilizing scRNA-seq and AI, researchers can reveal the complex interactions between LR cancer cells and non-malignant stromal cells, further analyzing cellular composition, heterogeneity, and developmental trajectories, thereby systematically mapping the TME 118, 119. Zhou *et al.* discovered that Mucosal-associated invariant T cells can confer resistance to the combination therapy of lenvatinib and anti-PD1 antibodies in HCC via the TNF-TNFRSF1B pathway, as identified by scRNA-seq 120. Additionally, novel molecular typing and prediction models based on sequencing and machine learning have brought new hope for the precision and personalized treatment of malignant tumors. In a recent study, Zhang *et al.* employed a machine-learning-based approach to identify a panel of 13 signature genes that served as predictive biomarkers for lenvatinib response, achieving an area under the receiver operating characteristic curve of 0.86 121. The CRISPR system, a revolutionary genomic editing tool, is fundamentally changing cancer research paradigms and treatment modalities. It can comprehensively screen and precisely manipulate genes that drive tumor growth and resistance, opening up new possibilities for the development of more effective and personalized cancer therapies 122. Pan *et al.*'s research, using genomic CRISPR-Cas9 screening, identified LAPTM5 as a key factor of LR in HCC, with the role of LAPTM5 being to enhance the formation of autolysosomes 48.

RCD was also reported to contribute substantially to the development of drug resistance in other targeted therapy and immunotherapy for HCC, and corresponding therapeutic strategies were proposed. Sorafenib is another first-line targeted therapy for advanced HCC. Lai's study revealed that the resistance of EGFR-overexpressing HCC cells to sorafenib was linked to inadequate autophagic activation, and metformin administration could enhance sorafenib sensitivity and autophagy via the activation of AMPK 123. Elevated expression of fatty acid synthase (FASN) counteracted SLC7A11-related ferroptosis and therefore fostered resistance to sorafenib, and Orlistat, an inhibitor of FASN, could effectively improve the efficacy of sorafenib by promoting ferroptosis 124. Immune checkpoint inhibitors (ICIs), containing anti-PD-1 treatment, have emerged as promising immunotherapies for advanced HCC. Gao's research demonstrated that anti-PD-1 treatment elevated autophagy and the expression of Yes-associated protein 1 (YAP1), and Yap1 knockout enhanced the effectiveness of anti-PD-1 treatment by inhibiting autophagy in HCC 125. Zheng *et al.* showed that inhibition of phosphoglycerate mutase 1 (PGAM1) could enhance the efficacy of anti-PD-1 treatment in HCC by stimulating ferroptosis and the infiltration of CD8+ T-cells 126.

Despite the promising nature of studies regarding combination therapy or nanomaterials to boost the effectiveness of lenvatinib and reverse LR in HCC cell lines and animal models, few have been granted approval for clinical use. After conducting an exhaustive literature review and searching the ClinicalTrials.gov website, we identified one clinical trial, that focused on combating LR with the mentioned RCD modulators. It is a prospective clinical study aiming to test the safety and efficacy of lenvatinib in combination with gefitinib in people with lenvatinib resistant hepatocellular carcinoma. The inclusion criteria includes: (1) Unlimited gender, aged 18-75 years; (2) Meets American Association for the Study of Liver Diseases or European Association for the Study of the Liver clinical diagnostic criteria of HCC; (3) progressing after standard treatment; (4) Unresponsive or resistant to Lenvatinib; (5) Child-Pugh A or scored 7 B; (6) Eastern Cooperative Oncology Group performance status score <=1; (7) Platelet count >=60x10^9/L, Prothrombin time prolonged <=6 seconds. The results are as follows: following approximately 4 weeks of lenvatinib plus gefitinib therapy, 12 treated HCC patients exhibited a reduction in total tumor burden, as evidenced by MRI images, which was indicative of partial response. In addition to further investigating the molecular mechanism of LR, translating these insights into tangible clinical applications is a critical next step. Hence, corresponding clinical trials are urgently needed to test the safety and efficacy of these therapeutic strategies in real-world scenarios.

## Figures and Tables

**Table 1:** Drugs overcome LR via regulating RCD

Drugs	Target	Mechanism	Phase	NCT number	Refs.
Oxysophocarpine	FGFR1/AKT/mTOR	apoptosis	-	-	26
Dicoumarol	NQO1	apoptosis	-	-	28
XAV-939	IRF2/β-catenin	apoptosis	-	-	31
Metformin	AKT/FOXO3	apoptosis	-	-	101
SAHA	PTEN/PI3K/AKT	apoptosis	-	-	102
TMP269	FGFR	apoptosis	-	-	103
Sophoridine	VEGFR2/RAS/ERK	apoptosis	-	-	104
Elacridar	MDR1 and BCRP	apoptosis	-	-	105
Gefitinib	EGFR/PI3K/AKT	apoptosis	Post-market	NCT04642547	105
Copanlisib	PI3K/AKT	apoptosis	-	-	105
STM2457	METTL3/EGFR	apoptosis	-	-	40
Reserpine	METTL3/SMAD3	apoptosis	-	-	107
HCQ	-	autophagy	-	-	48
CQ	-	mitophagy	-	-	62
3-MA	-	autophagy	-	-	50
Olomoucine	BNIP3-AMPK-ENO2	mitophagy	-	-	63
CP	-	autophagy	-	-	111
Metformin	METTL3/PPARGC1A	ferroptosis	-	-	80
ZZW-115	circPIAS1/miR-455-3p/NUPR1	ferroptosis	-	-	86
AKT-IN3	GPX4/GRHL3/ PTEN/PI3K/AKT	ferroptosis	-	-	77
Solanum torvum	HO-1/GPX4	ferroptosis	-	-	76
Artesunate	TFRC	ferroptosis	-	-	78
DSF	DLAT, LIAS, CDKN2A, FDX1	cuproptosis	-	-	92

Abbreviations: LR: lenvatinib resistance; RCD: regulated cell death; HCQ: hydroxychloroquine; CQ: chloroquine; 3-MA: 3-Methyladenine; CP: Compound Phyllanthus urinaria; DSF: disulfiram.

## Abbreviations

ac4C: N4-acetocytidine
AI: artificial intelligence
BAMBI: bone morphogenetic protein and activin membrane-bound inhibitor
BNIP3: BCL2 interacting protein 3
ceRNA: competitive endogenous RNA
circRNAs: circular RNAs
CK2: casein kinase-2
CP: Compound Phyllanthus urinaria
CQ: chloroquine
CSC: cancer stem cell
DLAT: Dihydrolipoamide S-acetyltransferase
DSF: disulfiram
EMT: epithelial-mesenchymal transition
ENO2: enolase 2
ER: endoplasmic reticulum
EVA1A: eva-1 homolog A
FAM134B: family with sequence similarity 134, member B
FASN: fatty acid synthase
FDA: Food and Drug Administration
FGFR: fibroblast growth factor receptor
FOXA2: forkhead box protein A2
FOXO3: Forkhead box protein O3
FTH1: ferritin heavy chain 1
GPX: glutathione peroxidase
GRHL3: grainyhead-like transcription factor 3
HCC: hepatocellular carcinoma
HCQ: hydroxychloroquine
HO-1: heme oxygenase-1
ICD: immunogenic cell death
ICIs: immune checkpoint inhibitors
IRF2: interferon regulatory factor 2
JMJD8: Jumonji domain-containing protein 8
KPNB1: karyopherin subunit beta 1
LACTB: serine beta-lactamase-like protein
LAPTM5: lysosomal protein transmembrane 5
lncRNAs: long noncoding RNAs
LR: lenvatinib resistance
LT: liver transplantation
METTL: methyltransferase-like protein
miRNAs: microRNAs
NQO1: NAD(P)H:quinone oxidoreductase 1
Nrf2: nuclear factor erythrocyte 2-related factor 2
NRP1: neuropilin-1
NUPR1: nuclear protein 1
ORR: objective response rate
OS: overall survival
PABPC4: poly (A) binding protein cytoplasmic 4
PFS: progression-free survival
PINK1: PTEN-induced putative kinase 1
PGAM1: phosphoglycerate mutase 1
p-MYH9: phosphorylated non-muscle myosin heavy chain 9
PPARGC1A: peroxisome proliferator-activated receptor-gamma coactivator-1α
RCD: regulated cell death
RFA: radiofrequency ablation
ROS: reactive oxygen species
SBRT: stereotactic body radiotherapy
scRNA-seq: single-cell RNA sequencing
SD: stable disease
SLC7A11: solute carrier family 7 member 11
STOML2: stomatin-like protein 2
STX6: syntaxin-6
TACE: transcatheter arterial chemoembolization
TCA: tricarboxylic acid
TCM: traditional Chinese medicine
TFEB: transcription factor EB
TKI: tyrosine kinase inhibitor
TME: tumor microenvironment
TMX2: thioredoxin related transmembrane protein 2
TR: transferrin receptor
TTP: time to progression
USF2: upstream stimulatory factor 2
USP22: ubiquitin-specific protease 22
VEGFR: vascular endothelial growth factor receptor
YAP1: Yes-associated protein 1
3-MA: 3-methyladenine
